# Seroprevalence screening for the West Nile virus in Malaysia’s Orang Asli population

**DOI:** 10.1186/s13071-014-0597-0

**Published:** 2014-12-17

**Authors:** Suria Marlina, Siti Fatimah Muhd Radzi, Rafidah Lani, Khor Chee Sieng, Nurul Farhana Abdul Rahim, Habibi Hassan, Chang Li-Yen, Sazaly AbuBakar, Keivan Zandi

**Affiliations:** Tropical Infectious Disease Research and Education Center (TIDREC), Department of Medical Microbiology, Faculty of Medicine, University Malaya, Kuala Lumpur, Malaysia

**Keywords:** West Nile virus, *Culex* mosquitoes, Malaysia’s Orang Asli, Seroprevalence, Migratory birds, Vector

## Abstract

**Background:**

West Nile virus (WNV) infection is an emerging zoonotic disease caused by an RNA virus of the genus *Flavivirus.* WNV is preserved in the environment through cyclic transmission, with mosquitoes, particularly *Culex* species, serving as a vector, birds as an amplifying host and humans and other mammals as dead-end hosts. To date, no studies have been carried out to determine the prevalence of the WNV antibody in Malaysia. The aim of this study was to screen for the seroprevalence of the WNV in Malaysia’s Orang Asli population.

**Methods:**

Serum samples of 742 Orang Asli were collected in seven states in peninsular Malaysia. The samples were assessed to determine the seroprevalence of WNV immunoglobulin (Ig)G with the WNV IgG enzyme-linked immunosorbent assay (ELISA) method. For each individual, we documented the demographic factors. Anti-dengue and anti-tick-borne encephalitis virus IgG ELISA were also performed to rule out a cross reaction. All statistical analyses were performed using the GraphPad Prism 6 (GraphPad Software, Inc.); p values of less than 0.05 were considered significant.

**Results:**

The serosurvey included 298 men (40.16%) and 444 women (59.84%) of Malaysia’s Orang Asli. Anti-WNV IgG was found in 9 of the 742 samples (1.21%). The seroprevalence was 0.67% (2 of 298) in men and 1.58% (7 of 444) in women. The presence of anti-WNV IgG was found not to be associated with gender but, however, did correlate with age. The peak seroprevalence was found to be 2.06% (2 of 97) in individuals between 30 to 42 years of age.

**Conclusions:**

No previous studies have examined the seroprevalence of the WNV antibody in the human population in Malaysia, and no clinical reports of infections have been made. Screening for the WNV seroprevalence is very significant because of many risk factors contribute to the presence of WNV in Malaysia, such as the abundance of *Culex* mosquitoes as the main vector and a high degree of biodiversity, including migratory birds that serve as a reservoir to the virus.

## Background

The WNV is a member of the virus family Flaviviridae, which belongs to the Japanese encephalitis virus (JEV) serogroup of flaviviruses and is closely associated with other human pathogens such as dengue virus (DENV), yellow fever virus (YFV) and tick-borne encephalitis virus (TBEV) [1]. The flaviviruses are positive sense, single-stranded RNA viruses [1]. Murray Valley encephalitis viruses (MVEV), St. Louis encephalitis virus (SLEV) and Usutu virus (USUV) are also included in the JEV serogroup [1,2]. The WNV species also contains the Kunjin virus (KUNV) subtype that is endemic in Australia and Malaysia [3]. The flaviviruses of the JEV serocomplex are the prominent cause of arboviral encephalitis in vertebrate hosts, including humans [2].

Phylogenetic lineage studies show that approximately 1000 years ago, WNV emerged as a distinctive virus and had developed into two distinct lineages [4]. Lineage 1 was found to be the source of epidemic transmission in Africa and throughout the world, whereas lineage 2 was discovered in horses in sub-Saharan Africa and Madagascar [5]. The West Nile virus was first isolated in a woman in the West Nile district of Uganda in 1937 [6]. The first recognition of WNV in the Western Hemisphere occurred in 1999 in New York City [6,7], apparently by transference from infected humans, birds or mosquitoes [8]. This emergence of WNV in North America marked the first time that this virus had been identified outside the Eastern Hemisphere [9]. It appears clear that the source of the WNV strain detected in New York City originated in the Middle East [10].

In 1957, in an outbreak amongst aged patients in Israel, the virus was recognized as a cause of severe human meningitis or encephalitis (inflammation of the brain and spinal cord) [7]. It was usually related with asymptomatic, self-limiting childhood infections in humans [11]. Since then, the disease has spread through much of the world including Africa, Europe, the Middle East, central Asia and recently, North America. It has been detected in humans, animals and mosquitoes in all of these regions [11]. In 2000, the epizootic extended to 12 states and the District of Columbia [12], and WNV can now be found in many avian and mosquito species throughout North America [13,14]. From 1999 to 2010, more than 2.5 million people were infected, with over 12,000 reported cases of encephalitis or meningitis and over 1,300 deaths [15].

The presentation of clinical illness in humans ranges from asymptomatic infection to viral syndrome to neurologic disease [16]. In the epidemic of WNV infection, it is estimated that 80% of infections are asymptomatic and the other 20% present as a dengue-like viral syndrome with fever, headache, body aches and sometimes a skin rash on the trunk and swollen lymph glands [17]. It can be severe but is commonly self-limited, and less than 1% of cases lead to neuroinvasive disease such as encephalitis, meningitis or polio-like flaccid paralysis [16]. The incubation period may be as short as 2 days or as long as 14 days, and the mortality rate ranges from 4% to 15%, whereas patients with encephalitis and flaccid paralysis have a poorer prognosis [18].

WNV is a mosquito-borne flavivirus transmitted to humans by mosquitoes, primarily the *Culex* genus, in particular *Culex pipiens* that serve as a vector, birds that serve as intensifying hosts and humans or others mammals that serve as dead-end hosts [19-21]. Zoonotic WNV circulates in natural transmission cycles comprising mosquitoes and birds and a range of other vertebrates, such as horses and humans, are incidental hosts. WNV is maintained in mosquito populations through vertical transmission (adults to eggs); birds are an important reservoir of WNV in the environment; more than 200 species of birds in the United States were found to be infected [22]. Studies have shown that transmission between birds in the absence of mosquitoes can occur in a laboratory setting; however it is unknown whether this would be the case in the natural environment [22]. In prominent of dynamics pathogen transmission, the ecology activities of hosts can play an important role [23]. Host searching ecology, habitat affection and social communication are able to manipulate the possibility of contact with an infected host or the environment and thus construct hotspot of transmission [23-25]. Many species of vertebrates can be infected by these virus-carrying mosquitoes, including horses, which have a high mortality rate [26]. Humans usually experience a low level of viremia; however, transmission through organ transplantation and blood transfusion has been recorded [26]. A case of transmission through breastfeeding was reported, but the infant remained asymptomatic [26].

At present, no studies have been carried out in Malaysia regarding the seroprevalence of the WNV antibody in the human population, and no clinical reports of infections have been made. This could be due to undiagnosed or misdiagnosed subclinical infections or because the WNV has not yet reached Malaysia. The objective of this study was to screen for the prevalence of antibodies against WNV, particularly immunoglobulin (Ig)G, among Malaysia’s Orang Asli, the native people, who make up less than 1% of the Malaysian population. Most of them reside at the frontier of the jungle and rural areas (61%) and in the center of the jungle (37%), and only a small number of them are found in or close to an urban area (1%). Because dengue fever is endemic in Malaysia, a comparison with the anti-dengue IgG antibody result is also discussed to rule out cross reactivity.

## Methods

### Ethics statement

The study acknowledged distinctive consent from the Department of Orang Asli Development (JAKOA) and attained approval from the Ethics Committee of the University Malaya Medical Centre (UMMC) (MEC Ref.824.11). Contribution in this study was virtuously voluntary. The volunteers were given a briefing on the project and were allowed adequate time for consideration. Written informed consent was acquired. An additional agreement form was completed by the parents or guardian of volunteers below legal age (less than 18 years old). The samples were prepared with rigid obscurity. All volunteers had completed a given questionnaire to identify symptoms they had experienced and their lifestyle. All volunteers gave written consent for the use of blood samples after the samples were made anonymous.

### Sample collection

All blood samples were collected between September 2012 and February 2013 from 742 Orang Asli from seven states in peninsular Malaysia (Perak, Melaka, Pahang, Negeri Sembilan, Kelantan, Selangor and Johor states) with the assistance of a proficient medical assistant. Most of the Orang Asli were forest inhabitants. They were interviewed and given a standard questionnaire regarding demographic features, medical history and disclosure of potential risk.

### Anti-WNV serology

WNV IgG testing was done by WNV IgG capture DxSelect^TM^ELISA (Focus Diagnostics, Cypress, USA) according to the manufacturer’s instructions [5,27,28]. The serum samples were also tested for the presence of IgM of tick-borne encephalitis virus (TBEV) and dengue virus (DENV) —flaviviruses commonly found in Malaysia — so as to rule out cross reactivity. In this assay, polystyrene microwells were coated with recombinant WNV antigen. The tests were performed according to the manufacturer’s instructions. According to the manufacturer, the sensitivity was determined with the samples with expected positivity for IgG anti-WNV (vaccinated for flaviviruses or persons with past WNV infection). Serum samples were taken from the unvaccinated blood samples.

### Statistical analysis

Categorical data were assessed in two-way contingency table analyses using Fisher’s exact test. The correlation of age and reactivity of WNV infection were determined by means of the Spearman nonparametric correlation. All statistical analyses were performed using GraphPad Prism 6 (GraphPad Software, Inc.), and p values of less than 0.05 were considered significant.

## Results

**Figure 1:**
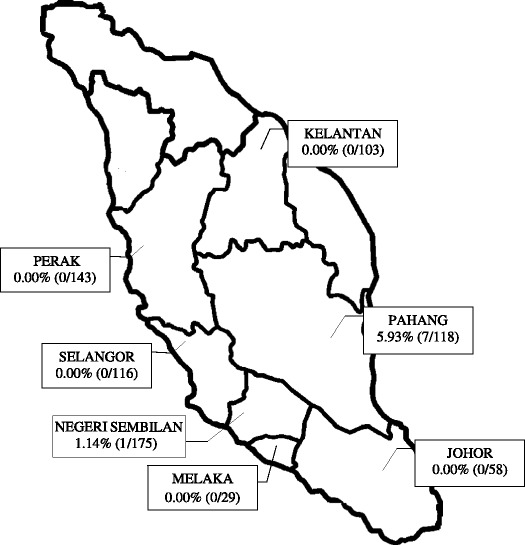
**Reactive serum of WNV in Peninsular Malaysia.** The figure indicated the distribution of reactive serum of WNV from the Malaysia’s Orang Asli collected throughout the study site in peninsular Malaysia.

An overview of the characteristics of the 742 serum samples are shown in Figure 1 (listed by state in Malaysia) and Table 1 (listed by age and gender). The study panel included 298 male volunteers with a median age of 13 and 444 female volunteers with a median age of 20.Overall, ages ranged from 3 to 90 years, with a median of 17. A total of 370 volunteers (49.86%) were 17 years or younger, and 372 volunteers (50.13%) were 17 years and older. Analysis by state indicated that Pahang had the highest percentage of reactivity to the anti-WNV IgG enzyme-linked immunosorbent assay (ELISA) test (5.93%), and five states (Selangor, Johor, Melaka, Kelantan and Perak) did not show the presence of WNV.

**Table 1:** **Seroprevalence of WNV according to age**

**GENERAL PROFILE**		**SEROPOSITIVE**	**SERONEGATIVE**	**TOTAL**	**% (95% CI)**
**GENDER**	MALE	2	296	298	0.67 (0.0002-0.0258)
	FEMALE	7	437	444	1.6 (0.0070-0.0329)
**AGE (YEARS)**	≤16	4	360	364	1.1 (0.0030-0.0282)
	17 to 29	2	169	171	1.18 (0.0005-0.0449)
	30 to 42	2	97	99	2.06 (0.0012-0.0766)
	43 to 55	1	63	64	1.58 (<0.0001-0.0927)
	56 to 68	0	33	33	0 (0.00-0.1239)
	≥69	0	11	11	0 (0.00-0.3002)

Table showed the seroprevalence of WNV following to the age-group among the Malaysia’s Orang Asli.

**Figure 2:**
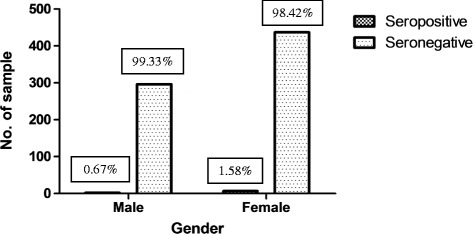
**Reactivity of WNV towards gender.** The figure presented that there is no correlation between the gender and the reactivity of samples of the WNV.

**Figure 3:**
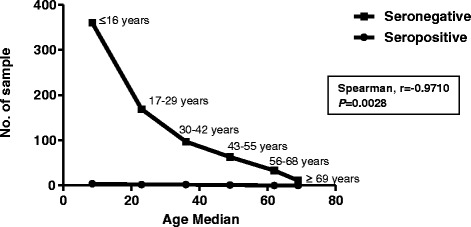
**Reactivity of WNV towards age.** The figure shows the correlation between the age group and the reactivity of samples of the WNV.

In general, anti-WNV IgG was discovered in 9 of 742 samples (1.21%). There was no significant difference in the seroprevalence between the male (0.67%; 95% confidence interval [CI], 0.0002 to 0.0258) and female (1.58%; 95% CI, 0.0070 to 0.0329; *P* = 0.3270) groups (Table 1). The highest rates of reactivity to anti-WNV IgG ELISA were 3.13% (95% CI, <0.0001 to 0.1711) for men between 43 and 55 years age and 2.74% (95% CI, 0.0018 to 0.1002) for women between 30 and 42 years of age (*P* = 1.000). A summary of serum prevalence by age and gender is presented in Table 2. At least 86 of the 742 samples (11.59%) exhibited cross-reactivity for anti-TBEV IgG and 1 of 742 (0.13%) for anti-dengue IgG, whereas 14 of the 742 samples (1.89%) were found to have cross-reactivity for all three types of viruses (Table 3). The study also showed that there was no significant difference regarding antibody prevalence and gender (Fisher’s exact test, *P* = 0.3270). The seroprevalence was 0.67% (2 of 298) in male subjects and 1.58% (7 of 444) in female subjects (Figure 2). However, as illustrated in Figure 3, the different age groups were associated with reactivity of the sample (*P* = 0.0028) (Spearman, r = −0.9710).

**Table 2:** **Prevalence serum of WNV regards to age and gender**

**AGE**	**MALE**		**FEMALE**	
	**SEROPOSITIVE**	**SERONEGATIVE**	**SEROPOSITIVE**	**SERONEGATIVE**
**≤16**	0	174	4	186
**17 to 29**	1	47	1	122
**30 to 42**	0	26	2	71
**43 to 55**	1	31	0	32
**56 to 68**	0	14	0	19
**≥69**	0	4	0	7
**TOTAL**	**298**		**444**	

Table presented the summary of prevalence serum of WNV regards to both age and gender of Malaysia’s Orang Asli.

**Table 3:** **Cross-reactivity of WNV against TBEV, DENV and all three types of viruses**

	**TBEV (%)**	**DENV (%)**	**TBEV AND DENV (%)**
**SEROPOSITIVE**	11.59	0.13	1.89
**SERONEGATIVE**	88.41	99.87	98.11

Table presented the exhibited cross-reactivity of WNV for TBEV, DENV and for all three types of viruses.

## Discussion

To date, no studies have been carried out in Malaysia regarding the seroprevalence of the WNV antibody in the human population, and no clinical reports of infections have been made. Screening for WNV seroprevalence is necessary and important because of many risk factors for the presence of WNV in Malaysia, such as the abundance of *Culex* mosquitoes as the main vector and the appropriate climatic situation for the propagation of mosquitoes and migratory birds that serve as a reservoir for the virus [29]. This assertion was supported by a study that showed *Culex* mosquitoes are abundantly distributed across the states of Malaysia [30]. Moreover, preceding study on the behavior of host such as their nocturnal roosting patterns is significantly important in WNV transmission [23]. This natural behavior is expected to affect the contact rates with hunting mosquitoes. The abundance of host-seeking *Culex* mosquitoes may be higher in the forest canopy [25] so birds roosting may be visible to more mosquito bite [23]. In addition, previous study was done in north-western Italy proved that precipitation and warm temperature is strongly related to the WNV transmission [31]. Even though, the warm temperature does put in the diverge effect on vector competence, still it does influence in the abundance of adult mosquitoes and thus providing more potential vectors for WNV [32].

This study is the first such to be conducted in Malaysia. We screened 742 serum samples from Malaysia’s Orang Asli from seven states in peninsular Malaysia, and the results suggest that WNV has a low prevalence in Malaysia (1.21%, or 7 of 742). Although it is not considered endemic, a prolonged study should be done. In 2011, a study conducted among captive birds to determine the presence of the WNV antibody was carried out in four selected areas in Selangor and had showed 4.41% seropositivity rate [33]. As for the WNV transmission cycle, birds and *Culex* species mosquitoes are their principal vertebrate and vector hosts, respectively, and all are spread by migratory birds [20]. It is believed that birds infected with more virulent epidemic strains of the virus are too sick to migrate; thus, only birds infected with less virulent viral strains can make the long journey [20]. As for WNV vector competence, hybrid populations of *Culex* species mosquitoes produced from cross mating lines exhibited that hybridization has significant impact on WNV infection [34], dissemination and transmission which could be due to the environmental or anthropogenic changes [35].

We discovered that the cross-reactivity in IgG antibodies of WNV and DENV (0.13%) was lower than with TBEV (11.59%) [36]. The lower percentage of DENV IgG antibodies obtained from the serosurvey suggested that most of the Orang Asli people were less infected with WNV and DENV, though DENV is consider to be endemic in Malaysia compared to TBEV, because *Aedes aegypti* and *Culex pipiens* are the main vector for DENV and WNV, respectively, which can be found abundantly in rural areas but not in the forest [37,38]. This could have an effect on the seroreactivity of the samples because the volunteers were forest-dwellers.

Due to some limitation in this study, neutralisation tests were not executed for the confirmation of positive WNV antibodies by ELISA tests. According to the previous study, patients with positive WNV infection by ELISA were also confirmed to be positive by neutralisation tests [4]. The WNV IgG resulted by an ELISA is a dependable marker of the presence of neutralizing antibodies against WNV when assuming the absence of previous history of other flavivirus vaccinations or infections in an individual. The ELISA fulfilled the necessities of this study, since the main objective was to determine the prevalence of WNV IgG among the Malaysia’s Orang Asli population, even though the risk that the subjects either had susceptibility or were indicating cross-reactive antibodies and considering the likelihood that the virus had been blank from the blood at the time the samples have been engaged.

Because there was doubt about cross-reactivity with other viruses, the IgG ELISA test was performed on two other flaviviruses, TBEV and dengue virus. However, only 86 of 742 samples (11.59%) exhibited cross-reactivity for anti-TBEV IgG and only 1 of 742 samples (0.13%) for anti-dengue IgG, whereas 14 of the 742 samples (1.89%) were found to have cross-reactivity for all three types of viruses.

Another study showed that the seropositivity of WNV did not correlate with age and gender [4], nevertheless we found that age did correlate with the incidence of WNV infection; the highest rate of reactive serum samples was in the adult age group between 30 and 42 years of age, likely because people from this age group participate in a lot of outdoor activities such as hunting and working in the forest. Furthermore, there were fewer volunteers of 50 years and above of age in our study compared to other age groups. Moreover, in another study, it was found that seropositive persons were likely to be older than seronegative ones, but that age did not persist as an autonomous risk factor for the infection [5].

Although WNV is not endemic, our study demonstrates that the presence of WNV in several states in peninsular Malaysia could be an early warning of transmission of the virus. Transmission of the virus will be very difficult to prevent and control because the public health infrastructure is insufficient to cope with vector-borne and zoonotic diseases [20]. In the absence of a specific treatment or vaccine, efforts at preventing transmission should focus on vector breeding sites and public education. This issue is deserving of further study, as this is the first study to ascertain the presence of antibodies against WNV in Malaysia’s Orang Asli.

## Conclusions

No previous studies have examined the seroprevalence of the WNV antibody in the human population in Malaysia, and no clinical reports of infections have been made. Screening for the WNV seroprevalence is very significant because many risk factors contribute to the presence of WNV in Malaysia, such as the abundance of *Culex* mosquitoes as the main vector and a high degree of biodiversity, including migratory birds that serve as a reservoir to the virus.

## Abbreviations

WNV: West Nile virus
RNA: Ribonucleic acid
Ig: Immunoglobulin
ELISA: Enzyme-linked immunosorbent assay
JEV: Japanese encephalitis virus
DENV: Dengue virus
YFV: Yellow fever virus
TBEV: Tick-borne encephalitis virus
MVEV: Murray Valley encephalitis virus
SLEV: St. Louis encephalitis virus
USUV: Usutu virus
KUNV: Kunjin virus
JAKOA: Department of Orang Asli Development
UMMC: University Malaya Medical Centre
